# Morphological and Optical Characterization of NRAS‐Mutant Melanoma Cells and Primary Melanocytes via Quantitative Phase Imaging With Digital Holographic Microscopy

**DOI:** 10.1002/cbf.70156

**Published:** 2026-01-04

**Authors:** Ayah A. Farhat, Yazan A. Almahdi, Fatima Z. Alshuhani, Besa Xhabija

**Affiliations:** ^1^ Department of Natural Science, College of Arts, Sciences and Letters University of Michigan–Dearborn Dearborn Michigan USA

**Keywords:** cell morphology, clustering, holographic microscopy, melanocytes, melanoma, NRAS mutation, optical parameters, PCA, SK‐MEL‐2, t‐SNE

## Abstract

Early detection of melanoma, a highly variable and aggressive form of skin cancer, is crucial for improving patient outcomes. It is essential to distinguish malignant cells from normal melanocytes, and therefore, label‐free imaging methods that can do so are needed. Given the genotoxic effect of UV radiation, these mutations are numerous and affect many genes, including NRAS; therefore, therapeutic strategies can be directed toward these recurrent mutations. The aggressive nature of NRAS‐mutant melanoma contributes to poor patient prognosis, highlighting the need for early diagnosis. This study utilizes quantitative phase imaging (QPI) with digital holographic microscopy (DHM) to differentiate the morphology of NRAS‐mutant SK‐MEL‐2 cells from melanocytes using holographic microscopy; dimensionality reduction techniques, including Principal Component Analysis (PCA),t‐distributed stochastic neighbor embedding (t‐SNE); and clustering techniques, including K‐Means Elbow Plots and Hierarchical Clustering Dendrograms. The results demonstrated distinct morphologies and geometries between melanocytes and SK‐MEL‐2 cells, indicating that QPI with DHM can serve as a label‐free tool for identifying optical biomarkers in melanoma.

## Summary

Melanoma is a deadly skin cancer that can be difficult to distinguish from normal pigment‐producing cells. This study uses digital holographic microscopy and advanced data analysis to uncover specific morphological and optical differences between healthy melanocytes and NRAS‐mutant melanoma cells. Our findings reveal that optical properties are among the most important features separating the two cell types. These results highlight the potential of holographic microscopy as a label‐free diagnostic tool and provide a foundation for developing new biomarkers to improve early detection of NRAS‐driven melanoma.

## 1 Introduction

The incidence of melanoma, a severe form of skin cancer originating from the pigment‐producing melanocytes in the epidermis, has been rising steadily in the United States, with rates having more than doubled in the past three decades [1, 2, 3]. Despite melanoma constituting only 4% of skin cancer cases, it accounts for 75% of skin cancer‐related deaths [2]. If caught early, however, melanoma has an ~94% survival rate [4], highlighting the necessity of early diagnosis and treatment. Treatment necessitates the differentiation of melanoma from healthy melanocytes, which may be facilitated by the use of a wide range of biomarkers.

Determining the morphological differences between healthy melanocytes and melanoma cells helps to elucidate potential properties important to defining cancerous states [5]. Specific morphological biomarkers may help predict and spot early progression into a cancerous state [6]. Melanoma diagnoses today heavily rely on visual biomarkers, where the clinician examines for abnormal pigmentation on the patient and may perform a biopsy to further characterize the condition [7]. The tissue may subsequently be examined for optical thickness, immunohistochemical markers, specific DNA mutations, or a combination of biomarkers [8]. Later stages of melanoma, however, may be characterized by varying optical thickness due to metastasis, complicating the diagnosis [9, 10]. It is, therefore, critical to have an arsenal of biomarkers present to improve accurate diagnosis.

In order to better define melanocytes from melanoma, cellular morphology may be characterized via holographic microscopy. Holographic microscopes image cells via minimally invasive means, aiding in the differentiation between healthy and cancerous cells [11, 12, 13]. In fact, it has been utilized to characterize cellular structures such as area, optical thickness, and perimeter length, as well as cellular behavior, such as the contraction of embryonic cardiomyocytes, red blood cell membrane fluctuations, and changes to cell volume [13, 14, 15, 16, 17]. This technique is based on the principle of quantitative phase imaging (QPI), in which variations in the phase of light passing through a specimen are measured to reconstruct cell morphology and optical thickness in a label‐free manner [18]. When implemented using digital holographic microscopy (DHM), QPI enables real‐time, noninvasive visualization of live cells, making it well‐suited for studying cancer cell behavior and identifying morphological biomarkers [19]. Holographic microscopy, as a result, may be used as a vital tool in characterizing both melanocytes and melanoma cells by highlighting differences in morphology and behavior.

Morphological data may be subsequently interpreted with dimensionality reduction techniques, including Principal Component Analysis (PCA) and t‐distributed stochastic neighbor embedding (t‐SNE) [5, 20]. That is, a large data set could be simplified to highlight the relative variability between the two cell lines. While both PCA and t‐SNE are useful in simplifying data, t‐SNE maintains the local structure of the data and therefore helps isolate different classes within a single data set, hence it has even been incorporated in molecular biology settings [21]. Moreover, clustering techniques, including K‐Means Elbow Plots via the Random Forest Model and Hierarchical Clustering Dendrograms, also provide significant insights into the differences between melanocytes and melanoma cells by dividing morphological data points into clusters based on similarities [22]. Such analyses would help to potentially identify any outliers that differ from the observed patterns as well as identify similarly related characteristics. As such, these methods may be utilized to further characterize and distinguish melanoma cells from healthy melanocytes.

Techniques such as QPI, and in particular DHM, allow for a label‐free and a minimally invasive visualization of live cells. It does so by reconstructing the maps of optical path length that reflect variations in refractive index, thickness, and dry mass [14, 23, 24, 25, 26, 27]. Measurements of these parameters can potentially serve as biophysical indicators of cell growth, proliferation, division, and death. Interestingly, translation of these parameters to cancer models has been applied quite successfully [25, 27, 28, 29, 30, 31].

Recently, the adoption of deep learning–based segmentation methods, such as Cellpose, has enabled researchers to perform quantitative analysis of cell morphology from label‐free brightfield or phase‐contrast images through high‐throughput image segmentation. This process depends on intensity contrast and does not provide direct measurements of biophysical parameters [32, 33]. This limitation of AI‐based tools can be addressed by DHM, which adds value by providing intrinsic, quantitative phase information without the need for labeling or causing phototoxicity [17, 26]. This allows for real‐time monitoring of living cells over long periods of time. The extraction of optical metrics along with geometric features by DHM offers increased sensitivity to cellular states important for tumor progression and treatment response [34, 35, 36].

In this study, we compared primary epidermal melanocytes to the malignant melanoma cell line SK‐MEL‐2 using holographic microscopy via the HoloMonitor M4, dimensionality reduction techniques, and clustering analyses. The SK‐MEL‐2 cell line is characterized by a mutated *NRAS* gene, which is involved in the regulation of cell division [37, 38]. Poor patient prognosis of *NRAS*‐mutant melanoma may be partly attributed to its aggressive nature, hence the need for early diagnosis [38, 39]. In an attempt to overcome this gap, our study aims to further clarify morphological differences between healthy melanocytes and *NRAS*‐mutant melanoma. Further identification of differences between the two cell lines may aid in the development of biomarkers to better diagnose *NRAS*‐mutant melanoma. To our knowledge, no other study has compared the morphology of melanocytes and SK‐MEL‐2 cells, shedding light on the importance of this research. By analyzing morphological differences between melanocytes and melanoma, significant variations between the two cell lines may be captured to better define *NRAS*‐mutant melanoma, providing a route to develop targeted therapies.

## 2 Materials and Methods

### 2.1 Cell Culture and Holographic Imaging

Primary epidermal melanocytes (HEMa, ATCC PCS‐200‐013) were cultured in a medium consisting of Dermal Cell Basal Medium (ATCC PCS‐200‐030) and the Adult Melanocyte Growth Kit (ATCC PCS‐200‐042) at 37°C with 5% CO_2_. The medium consisted of the following components: 5 µg/mL rh Insulin, 50 µg/mL ascorbic acid, 6 mM l‐glutamine, 1.0 µM epinephrine, 1.5 mM calcium chloride, 0.2% peptide growth factor (proprietary formulation), and 1% M8 supplement (proprietary formulation). The melanoma cell line SK‐MEL‐2 (ATCC HTB‐68) was maintained in RPMI 1640, GlutaMAX supplement (Thermo Fisher), along with 10% fetal bovine serum (ATCC) and 1% penicillin–streptomycin (Thermo Fisher) at 37°C with 5% CO_2_. In addition, the SK‐MEL‐2 cells were routinely tested for mycoplasma contamination using the MycoAlert Mycoplasma Detection Kit (Lonza), and were confirmed to be mycoplasma‐free during the course of the experiments. Both cell lines were seeded and imaged over 58 h via a HoloMonitor M4 holographic imaging microscope (Phase Holographic Imaging, Lund, Sweden). All of the recordings were performed with a HoloMonitor M4 system (Phase Holographic Imaging). The instrument uses a 635 nm laser for phase imaging and is equipped with a motorized XYZ stage, which was used to control imaging positions during the experiment. Images were captured with a 20× objective at a frame size of 1024 × 1024 pixels. The travel limits of the stage travel 100 × 70 × 10 mm, which allowed for stable multiposition imaging. The laser unit remained outside the incubator as recommended by the manufacturer to maintain temperature stability. Cells were plated in standard 24‐well tissue‐culture plates (Corning, Cat. No. 3516). Just before the cells were placed in the holominotor, the plates were covered with HoloLids designed for Sarstedt plates (Phase Holographic Imaging), which provide a stable optical surface and reduce evaporation. These lids were used throughout all recordings to maintain uniform imaging conditions. Imaging was performed at 15‐min intervals in each well, 30 frames each, via the HoloMonitor M4. A total of 97 images (frames) of primary melanocytes and 73 images of SK‐MEL‐2 cells were analyzed after quality filtering. From these frames, the App Suite segmentation identified 21,908 melanocyte measurements and 21,908 SK‐MEL‐2 measurements that passed all inclusion criteria. These counts represent all segmented cells obtained from the three biological replicates per group. Frames containing debris, poor focus, or segmentation errors were removed prior to analysis. The App Suite Cell Imaging software associated with the HoloMonitor M4 completed Guided End‐Point Assays (Cell Quality Control) and In‐Depth Assays (Cell Morphology) upon analysis of these images in order to elucidate cell structure. We placed the HoloMonitor M4 system inside a standard CO_2_ incubator (Thermo Fisher Scientific) during all imaging sessions. Temperature and gas conditions were maintained at 37°C and 5% CO_2_ using the HoloMonitor stage‐top incubation chamber (Phase Holographic Imaging), which provides a stable environment for long‐term live‐cell recording. All images were acquired and analyses were performed using the HoloMonitor App Suite, version 4.0 (Phase Holographic Imaging).

### 2.2 2D, Pseudo‐3D Holographic Microscopy

The HoloMonitor M4 system captured images of the cultured melanocytes and SK‐MEL‐2 cells at 15‐min intervals in each well, 30 frames each, over the course of 58 h. The color palette of the captured 2D holographic images was altered from a grayscale to a rainbow palette to improve visual appeal. The colors applied across the images are relative to cell height, and the growth medium, which served as the baseline of comparison, is visualized in black. The scale ranges from 0 to 16 µm, with white/red colors reflecting greater elevations and blue/black colors reflecting lower elevations.

The single‐cell pseudo‐3D images of the melanocytes and SK‐MEL‐2 cells were developed through the “In‐Depth Analysis: Single Cell Tracking” package of the HoloMonitor M4's App Suite Cell Imaging software. Here, the 2D holographic images developed earlier were converted to their respective pseudo‐3D structures under the “Viewer Options” panel. The aforementioned rainbow color palette was also applied to the pseudo‐3D images to maintain consistency with the 2D images. Similarly, the color palette reflects changes in optical thickness relative to the growth medium, which served as the baseline. The thickness scale ranges from 0 to 16 µm. In order to measure the length, width, and peaks of the melanocyte and SK‐MEL‐2 cells, the “Measure” feature under the “Viewer Options” panel was utilized. The distance across the cells is indicated by a blue bar, and the respective measurements taken may be viewed in the graph at the bottom right of the image, titled “Profile Analysis.”

In the interest of this study, the measurements referred to as optical thickness represent values reconstructed by the HoloMonitor M4 from phase‐shift information in the holographic images. These values correspond to the apparent thickness of the cells, as determined by the differences in refractive index between the cells and the surrounding medium, as calculated by the instrument software [15, 40]. Because the actual refractive index of each cell type was not directly measured, the reported thickness values should be interpreted as relative optical parameters rather than absolute physical dimensions [41].

The Holomontor App suite was utilized to process quantitative phase images. We identified cells from the phase map using the built‐in threshold method, and the sensitivity and background threshold sliders were adjusted until the masks followed the visible outline of each cell. The minimum object size was tuned to avoid splitting long melanocyte branches or merging neighboring SK‐MEL‐2 cells. Frames with poor focus, debris, or very crowded fields were removed. Once the cell masks were set, the software calculated area, perimeter, convexity, eccentricity, and texture values directly from the mask. Optical thickness and optical path length were obtained from the phaseshift using the standard App Suite conversion based on the default refractive index settings (cell 1.38, medium 1.34). Optical volume was determined by integrating the reconstructed height over the cell area. To ensure the measurements were reliable, the masks were checked by eye during setup, and frames flagged by the software as unsuitable were excluded. Both melanocytes and SK‐MEL‐2 cells were processed using the same imaging and segmentation settings, so the comparisons reported here reflect consistent handling of both groups.

### 2.3 K‐Means Clustering and Hierarchical Clustering Dendrogram Generation

The data set used in this study contains various morphological and optical properties of melanocytes and SK‐MEL‐2 cells, including area, centroid positions, eccentricity, hull convexity, irregularity, optical path length, optical thickness, optical volume, phaseshift, roughness, shape convexity, and texture properties.

The initial data set was inspected for any missing values and inconsistencies. Features containing “max” and “min” in their names were filtered out to focus on the average (avg) features. The remaining data were then normalized to ensure that all of the features contributed equally to the analysis.

K‐Means clustering was performed to identify natural groupings within the data and assess the separation between melanocytes and SK‐MEL‐2 cells [42, 43, 44]. The Elbow Method was used to estimate the optimal number of clusters by plotting the total within‐cluster sum of squares against the number of clusters [45, 46]. The optimal number of clusters was found to be around 2. K‐Means clustering was then performed with two clusters, and the results were visualized using PCA. Hierarchical clustering was performed to visualize the relationships between the samples. The hierarchical clustering was done using the Ward's method, which minimizes the total within‐cluster variance [47, 48]. A dendrogram was created to visualize the hierarchical relationships between the samples, with clusters color‐coded to indicate the two cell types.

All data preprocessing and visualization were performed using R (version 4.0.5), with ggplot2 used to create visualizations and fact extra for clustering analysis and visualization. Clustering results were validated by visual inspection of the PCA and t‐SNE plots, as well as the dendrogram from hierarchical clustering.

### 2.4 PCA and t‐SNE Plot Generation

PCA and t‐SNE plots were developed in order to visualize the overall variability between the melanocytes and SK‐MEL‐2 cells. The data sets analyzed consisted of the parameters under study, including morphological, optical, geometric, positional, phaseshift, roughness, and texture values. For the PCA plot, the melanocyte and SK‐MEL‐2 data sets were separately standardized using the StandardScaler function from the scikit‐learn library in Python [49, 50]; upon standardization, each feature had a mean of 0 and a standard deviation of 1 [51]. Standardization is crucial in assuring each variable has an equal contribution to the PCA, which visualizes the overall variance of the data set. Any variable with a different scale of variance, therefore, would skew the analysis. PCA was then completed separately on the two standardized data sets using the first two principal components, which captures the variance across two axes. The results were then visualized using matplotlib, with the melanocytes represented by the color blue and SK‐MEL‐2 cells with orange. The resulting scatterplot enables a comparison of the variance within and between the melanocytes and SK‐MEL‐2 cell data. Identified patterns may indicate overarching differences between the two cell lines, allowing for greater characterization and thus potential for biomedical applications.

With respect to the t‐SNE plot, the standardized melanocyte and SK‐MEL‐2 cell data sets were combined into one data set. t‐SNE analysis was then performed with the TSNE class from the scikit‐learn library in Python, using two components [52]. Furthermore, for reproducibility, the algorithm ran with a random state of 42. Similar to the PCA plot, the results were plotted on a scatterplot using matplotlib, with the melanocytes represented in blue and the SK‐MEL‐2 cells in orange. The t‐SNE plot visualized the variance between the melanocyte and SK‐MEL‐2 characteristics under study, revealing local clusters and patterns to a greater degree than the PCA plot, which highlights global data patterns.

### 2.5 Violin Plot Generation for Each Parameter Under Study

Morphological, optical, positional, phaseshift, roughness, and texture cell data were collected over 58 h at 15‐min intervals, and the average cell data for each parameter was measured based on three biological replicates for both melanocytes and SK‐MEL‐2 cells. The measurements were recorded at each time frame via the HoloMonitor M4 software. Correspondingly, R (version 4.4.0) was utilized to generate violin plots as a means to compare the average differences between the melanocytes and SK‐MEL‐2 cells. The ggplot2 and tidyr libraries were implemented to create the plots and organize (i.e., tidy) the data, respectively. A combined data frame was then created via the average cell data at each frame, and the interquartile range for each parameter was calculated. The generated violin plot for each parameter has an *x*‐axis labeled with the cell line under study and a *y*‐axis indicating the parameter under study. The outline of the violin plots represents the probability distribution of the data, and the box plot within each plot indicates the median of the data and the interquartile range. Melanocytes and SK‐MEL‐2 cells were distinguished by the colors blue and orange, respectively. To evaluate the statistical significance of the differences in each of the parameters tested, the Mann–Whitney *U* test was employed given its suitability for the non‐normal data distribution. It was performed using the wilcox.test function in R (version 4.4.0), which calculated *p* values to compare a given parameter between the melanocytes and SK‐MEL‐2 cells.

### 2.6 Generation of the Ranked Feature Importance Chart Based on Random Forest Model

A Ranked Feature Importance Chart was developed to characterize the variables most significant in distinguishing between the melanocytes and SK‐MEL‐2 cells. The initial data set was inspected for any missing values and inconsistencies. To identify the most important features for distinguishing between melanocytes and SK‐MEL‐2 cells, a Random Forest model was employed. The feature importance was calculated based on the Mean Decrease in Accuracy and Mean Decrease in Gini. The Random Forest model was trained using the relevant predictors, excluding the irrelevant features and focusing on the average features. The response variable was the cell type (melanocytes or SK‐MEL‐2). The model was trained with the number of trees set to 500, and the number of variables tried at each split as sqrt (number of predictors) [53, 54, 55, 56].

The feature importance was extracted from the trained Random Forest model. The features were ranked based on their mean importance, focusing only on the average features. In turn, the feature importance was visualized using the ggplot2 library in R. A bar plot was created to rank the features based on their mean importance, highlighting the most significant features for distinguishing between Melanocytes and SK‐MEL‐2 cells. All data preprocessing and visualization were performed using R (version 4.0.5). The following R packages were used: randomForest for training the Random Forest model, ggplot2 for creating visualizations, and reshape2 for data manipulation. The statistical significance of the features was assessed based on their importance scores from the Random Forest model. Features with higher importance scores are considered more significant in distinguishing between the two cell types.

### 2.7 Measurement of Mean Cell Area, Volume, and Diameter

Mean cell area, volume, and diameter measurements were taken at 12‐h intervals over the course of 48 h, leading to the measurements at the following time periods: 0, 12, 24, 36, and 48 h. The mean cell area (µm^2^), volume (µm^3^), and diameter (µm) were obtained using the HoloMonitor M4 software. The mean cell area was determined geometrically from the number of pixels outlining each cell, while the mean cell diameter was approximated from the area using the standard area‐to‐diameter relation for a circle. This approach provides a consistent estimate of cell size despite deviations from circularity. In contrast, the mean cell volume was derived from the optical phaseshift of each cell, as implemented in the instrument's quantitative‐phase reconstruction algorithm, which assumes an average refractive index for the cells. Because the cellular refractive index was not directly measured, the reported volumetric and other phase‐based parameters represent relative optical measurements rather than absolute physical dimensions [57, 58]. Each time interval consisted of three biological replicates for both melanocytes and SK‐MEL‐2 cells. The mean cell area (µm^2^), volume (µm^3^), and diameter (µm) measurements were captured using the HoloMonitor M4 software. The HoloMonitor M4 software calculated the mean cell area by automatic measurements in the holographic images of each cell; the mean cell volume, by processing the cell hologram to reconstruct a three‐dimensional image; and the mean cell diameter, by taking the prior two measurements into account. The mean cell area, volume, and diameter results were represented using grouped bar graphs with standard deviation error bars. The bar graphs were generated in R using the ggplot2 and tidyr packages for visualization and data transformation, respectively. In order to create the bar graphs, the mean values and standard deviations for each time frame were taken into account. A data frame was created, with the data reshaped into long format for ggplot. The bar graph generated for each parameter consists of an *x*‐axis representing the hours the measurements were taken at and a *y*‐axis indicating the measured values. Standard deviation error bars were also included to display variations and potential significance in the results. Melanocytes and SK‐MEL‐2 cells were distinguished via different colors, blue and orange, respectively, on the bar graphs.

## 3 Results

### 3.1 2D, Pseudo‐3D Microscopy Reveal Overt Morphological Differences Between the Melanocytes and SK‐MEL‐2 Cells

**Figure 1:**
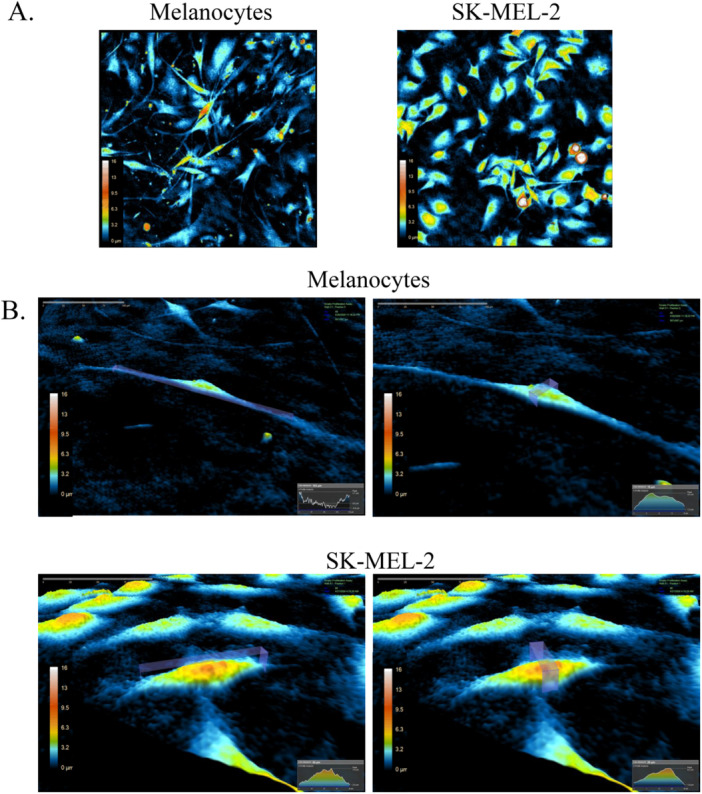
(A) 2D and (B) pseudo‐3D reconstructions of the same cells illustrating optical thickness measurements. In (B), the left images display the height (optical thickness) profile for each cell, while the right images show the corresponding width distribution. All subfigures have been enlarged and relabeled for improved visibility and clarity.

2D and pseudo‐3D microscopy highlighted the morphologies of the melanocytes and SK‐MEL‐2 cells, providing a means to determine distinguishing characteristics that may be further studied. For one, 2D microscopy is well‐suited for differentiating the two cell lines in terms of boundaries and shape. As demonstrated in Figure 1A, the melanocytes were characterized by a dendritic structure with multiple arms extending from their cell bodies, making up a great portion of the cell length. The SK‐MEL‐2 cells were characterized by polygonal, branched structures, though their dendrites were also notably shorter than those of the melanocytes. The 2D microscopy, overall, provided an overarching view of the overt morphological differences between the melanocytes and SK‐MEL‐2 cells, suggesting distinct morphologies.

Pseudo‐3D microscopy further elucidated cell structure by highlighting cell size in terms of length, width, and peak. The melanocyte observed in Figure 1B may be characterized by a width of 16 µm and a length of 163 µm, while the SK‐MEL‐2 cell under study may be characterized by a width of 26 µm and a length of 54 µm. Comparing these two cells, the melanocyte has notably longer branches than the SK‐MEL‐2 cells, as corroborated by the patterns observed in the 2D microscopy. In turn, the SK‐MEL‐2 cells were visibly more circular than the melanocytes. With respect to optical thickness, the melanocyte had a maximum peak of 4.7 µm, while the SK‐MEL‐2 cell had a maximum peak of 8.2 µm. The greater optical thickness of the SK‐MEL‐2 cell suggests a distinct cell structure from that of the melanocyte. These distinguishing patterns in the pseudo‐3D microscopy are largely representative of the differences observed between the two cell lines as a whole. Overall, 2D and pseudo‐3D microscopy provided insights into the overt melanocyte and SK‐MEL‐2 morphologies, which are suggested to be distinct from one another. The melanocytes were observed as containing multiple lengthy dendrites, and the SK‐MEL‐2 cells were observed with more polygonal boundaries and a greater optical thickness. Both melanocytes and SK‐MEL‐2 cells had elongated shapes with convex boundaries.

### 3.2 K‐Means Clustering and Hierarchical Clustering Dendrogram Demonstrate Usefulness of the Features Analyzed

**Figure 2:**
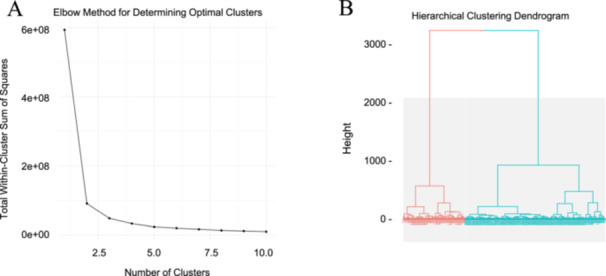
K‐Means Elbow Plot and Hierarchical Clustering Dendrogram comparing melanocytes and SK‐MEL‐2 cells using morphological, positional, geometric, phaseshift, roughness, and texture parameters. The K‐Means Elbow Plot (A) characterizes the optimal number of data clusters within the melanocyte and SK‐MEL‐2 data sets, as demonstrated by the “elbow” in the plot. The Hierarchical Clustering Dendrogram (B) visualizes the clustering of data points based on their similarity. The great height difference before breaking into two branches demonstrates a great difference between the two cell lines. These findings from the K‐Means Elbow Plot and Hierarchical Clustering Dendrograms suggest that the selected features highlight the differences between melanocytes and SK‐MEL‐2 cells. The plots were visualized using R (version 4.0.5). Analysis included 21,908 melanocyte measurements and 21,908 SK‐MEL‐2 measurements.

K‐Means clustering via the Elbow Method and Hierarchical Clustering Plots, as demonstrated in Figure 2, were used to divide the morphological data points, illustrated in Figures 4, 5, 6, 7, into clusters based on similarities in value. This clustering helps to potentially identify any anomalous data points that differ greatly from the typical patterns in the data. Both the K‐Means clustering and hierarchical clustering analyses demonstrate a clear separation between the melanocytes and SK‐MEL‐2 cells based on the selected features. The clustering results validate the feature selection process and highlight the effectiveness of the selected features in distinguishing between the two cell types. These findings provide strong evidence that the selected features capture the underlying biological differences between melanocytes and SK‐MEL‐2 cells, making them valuable for further analysis and potential diagnostic applications.

### 3.3 PCA and t‐SNE Plots Highlight Distinct Characteristics Between Melanocytes and SK‐MEL‐2 Cells

**Figure 3:**
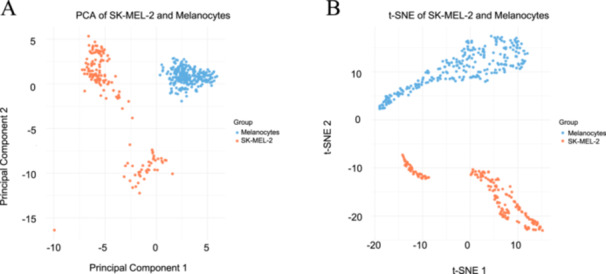
PCA and t‐SNE plots comparing melanocytes and SK‐MEL‐2 cells using morphological, positional, geometric, phaseshift, roughness, and texture parameters. The PCA plot (A) simplifies and separates the data across the first two principal components (PC1, PC2), visualizing variance while maintaining the global structure of the data. The t‐SNE plot (B) simplifies the high‐dimensional data and visualizes it across the two t‐SNE axes, maintaining the local structure of the data to a greater degree than the PCA plot. The clear separation between the melanocyte and SK‐MEL‐2 clusters in both the PCA and t‐SNE plots suggests significant differences between the two cell lines in terms of the parameters measured. The plots were generated in Python. Melanocytes are represented in the color blue, and SK‐MEL‐2 cells are visualized in the color orange. Analysis included 21,908 melanocyte measurements and 21,908 SK‐MEL‐2 measurements.

In order to investigate the similarities and variations of morphological traits between the melanocytes and SK‐MEL‐2 cells, the cells were graphed along a PCA plot and a t‐SNE plot, as seen in Figure 3. In Figure 3A, it is apparent in the PCA plot that the melanocyte data points are compactly clustered in one region, maintaining similar values along the Principal Component 1 (PC1) and the Principal Component 2 (PC2) axes, while the SK‐MEL‐2 cells exhibit a greater range along both the PC1 and PC2 axes. It is suggested, therefore, that there is a greater degree of similarity among the melanocytes in terms of their morphological characteristics, maintaining relatively similar profiles. This contrasts the much broader range encompassed by the SK‐MEL‐2 cells, thereby indicating a degree of variation within the SK‐MEL‐2 cell line itself. In addition, there is an overt separation between the melanocytes and SK‐MEL‐2 cells, with each cell line encompassing a distinct set of PC axes. Given the distinct separation between the melanocytes and SK‐MEL‐2 cells along these axes, it is suggested that their morphological differences are significantly different and thus may be used to differentiate between them. Overall, the PCA plot demonstrates distinct morphologies between the two cell lines and suggests a greater degree of homogeneity among melanocytes than among SK‐MEL‐2 cells.

The t‐SNE plot in Figure 3B visualizes high‐dimensional data onto a 2D plot while maintaining the local locations of the data points. It provides deeper insight into variations present between the two cell lines. In the plot, it is apparent that there is a separation between the melanocytes and SK‐MEL‐2 cell lines, suggesting that there is a great amount of dissimilarity in their morphological features. The t‐SNE plot also expands on the conclusions suggested by the PCA plot, as there is a degree of separation visualized between the melanocyte data points themselves. This separation suggests differences between the melanocytes as well, indicating that there is variation present even between the healthy cells. The t‐SNE plot, in total, corroborates the conclusions suggested by the PCA plot while also expanding on them.

### 3.4 SK‐MEL‐2 Cells Exhibit Greater Areas, Optical Properties, and Overall Distribution in Their Properties

**Figure 4:**
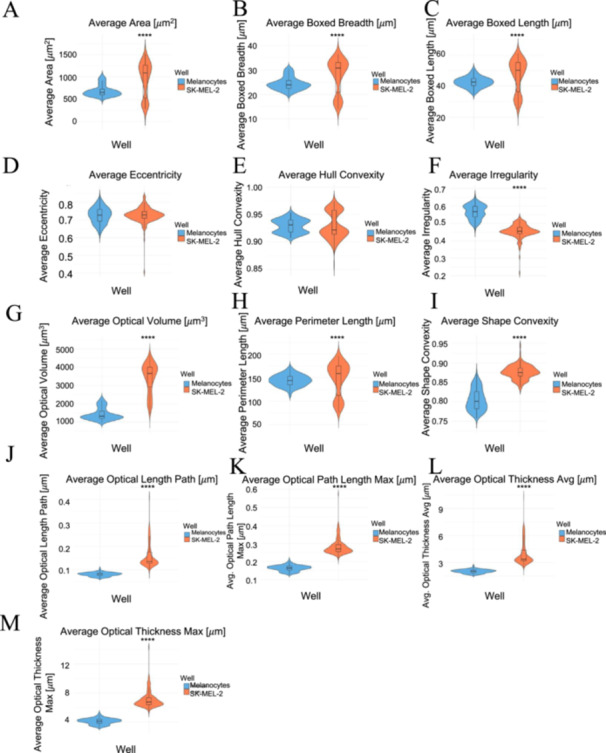
Violin plots of morphological features for melanocytes versus SK‐MEL‐2 melanoma cells. The melanocytes exhibited smaller, less polarized distributions of (A) average areas, (B) boxed breadth, and (C) boxed length, suggesting smaller areas with narrower sets of dimensions than SK‐MEL‐2 cells. The SK‐MEL‐2 cells exhibited similar values to the melanocytes, however, in (D) eccentricity and (E) hull convexity, suggesting similar elongation with convex shapes. The melanocytes exhibited greater (F) irregular boundaries with lower (G) optical volumes, (H) perimeter lengths, and (I) shape convexities. Additionally, melanocytes exhibited lower, more concentrated (J) optical path length avg, (K) optical path length max, (L) optical thickness avg, and (M) optical thickness max, indicating smaller thicknesses and path lengths than the SK‐MEL‐2 cells. Melanocytes are represented in the color blue, and SK‐MEL‐2 cells are visualized in the color orange. The plots were generated in R (version 4.4.0), and the statistical significance was indicated as **** for *p* < 0.0001 comparing the two cell lines. Analysis included 21,908 melanocyte measurements and 21,908 SK‐MEL‐2 measurements.

The violin plots in Figure 4 characterize the differences between the melanocytes and SK‐MEL‐2 cells regarding morphological features. With regard to the parameters of average area, boxed breadth, and boxed length (Figure 4A–C), SK‐MEL‐2 cells consistently displayed higher median values as well as greater value distributions. Melanocytes, on the contrary, exhibited smaller values overall that were also more concentrated around the median, suggesting that melanocytes typically have smaller areas with a more narrow set of dimensions.

However, eccentricity, hull convexity, and irregularity displayed different patterns in contrast to the above parameters (Figure 4D–F). With respect to eccentricity and hull convexity, both the melanocytes and SK‐MEL‐2 cells exhibited similar median values, suggesting that both cell lines are similarly elongated with convex shapes. Statistical significance was not observed compared to the melanocytes for these two parameters, suggesting similarity. Additionally, SK‐MEL‐2 cells had Irregularity values concentrated at lower values than the melanocytes, toward the median. These findings suggest that melanocytes have a more irregular circumference, while the SK‐MEL‐2 cells have more variation in their convexity.

The optical parameters of optical volume, perimeter length, and shape convexity displayed similar patterns to one another (Figure 4G–I); that is, the SK‐MEL‐2 cells exhibited higher median values for all three parameters, suggesting that SK‐MEL‐2 cells have greater volumes and perimeters with more convex cell outlines. With respect to optical volume and perimeter length, however, the SK‐MEL‐2 cells did have a greater variation in distribution compared to the melanocytes. These patterns suggest that SK‐MEL‐2 cells have more varied volumes and perimeters than melanocytes. Additional optical properties are illustrated in Figure 4J–M, including the average and maximum optical path length and optical thickness. SK‐MEL‐2 cells consistently display higher medians for all four optical parameters, suggesting that SK‐MEL‐2 cells have greater optical path lengths and thicknesses in comparison to the melanocytes.

The mean values were consistent with their respective median values across all of the parameters, as seen in Table 1. Hull convexity, however, deviated from this trend as although the melanocytes exhibited a higher overall median value, both melanocytes and SK‐MEL‐2 cells exhibited similar mean values. This discrepancy may be explained by the more polarized distribution of the SK‐MEL‐2 values as well as the presence of outliers compared to the melanocytes. Statistical significance using the Mann–Whitney *U* test was observed across all of the parameters except Eccentricity and Hull Convexity, suggesting potential to be used as a marker for differentiation. Overall, these findings suggest different external as well as internal morphological compositions, with SK‐MEL‐2 cells overwhelmingly having higher values for the majority of the parameters tested.

**Table 1:** Mean and Median Values of Violin Plots 3 dec points.

Feature	Melanocytes mean	Melanocytes median	SK‐MEL‐2 mean	SK‐MEL‐2 median
Area (µm²)	689.551	658.000	968.084	1092.000
Boxed breadth (μm)	24.407	23.900	27.874	30.800
Boxed length (μm)	42.548	42.600	47.056	50.200
Eccentricity	0.727	0.728	0.728	0.729
Hull convexity	0.929	0.931	0.929	0.922
Irregularity	0.566	0.568	0.456	0.456
Optical volume (µm³)	1419.416	1305.000	3412.317	3661.000
Perimeter length (μm)	144.317	144.000	147.758	159.000
Shape convexity	0.805	0.801	0.876	0.875
Optical path length Avg. (µm)	0.081	0.081	0.157	0.135
Optical path length Max. (µm)	0.163	0.164	0.282	0.270
Optical thickness avg. (µm)	2.020	2.030	3.927	3.370
Optical thickness max. (µm)	4.086	4.110	7.057	6.740
Boxed center position X (pxl)	517.379	516.000	580.114	595.000
Boxed center position Y (pxl)	544.786	542.000	594.281	571.000
Centroid position X (μm)	286.235	285.000	321.138	330.000
Centroid position Y (μm)	265.082	266.000	237.743	251.000
Peak position X (pxl)	517.831	516.000	581.060	596.000
Peak position Y (pxl)	544.922	543.000	594.287	570.000
Phaseshift avg.	0.127	0.128	0.247	0.212
Phaseshift std. dev.	0.046	0.046	0.079	0.080
Phaseshift sum	292.041	268.000	702.096	753.000
Roughness avg.	4.803	4.700	4.386	4.230
Texture clustershade	12.122	12.100	8.168	9.830
Texture clustertendency	10.763	10.700	11.814	12.700
Texture contrast	2.012	1.980	1.234	1.130
Texture correlation	0.673	0.680	0.790	0.828
Texture energy	0.050	0.050	0.059	0.059
Texture entropy	3.344	3.340	3.193	3.180
Texture max. prob.	0.104	0.103	0.120	0.121
Texture homogeneity	0.589	0.593	0.664	0.680

### 3.5 SK‐MEL‐2 Cells Reveal Greater Overall Variation in Their Geometric and Positional Parameters, With a Tendency for Higher Values

**Figure 5:**
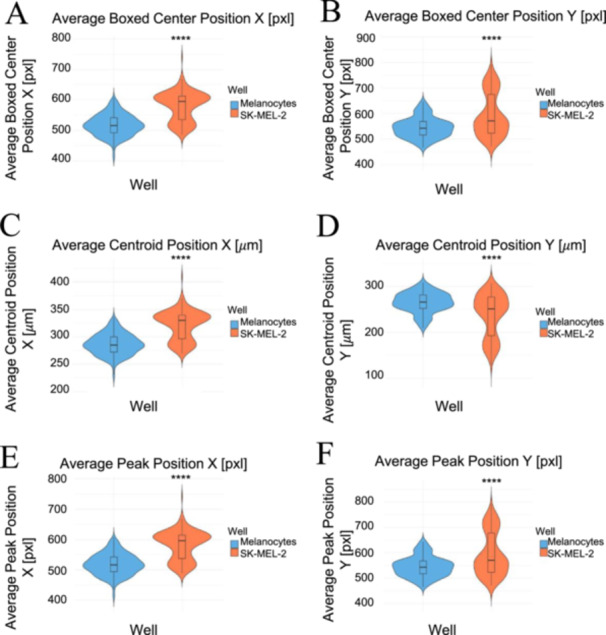
Violin plots of positional and geometric parameters for melanocytes versus SK‐MEL‐2 cells. The SK‐MEL‐2 cells exhibited more distributed values across the (A) boxed center position X and (B) Y; (C) centroid position X and (D) Y; and (E) peak position X and (F) Y, suggesting greater variation in the location of the geometric center and center of mass. Melanocytes are represented in the color blue, and SK‐MEL‐2 cells are visualized in the color orange. The plots were generated in R (version 4.4.0), and the statistical significance was indicated as **** for *p* < 0.0001 comparing the two cell lines. Analysis included 21,908 melanocyte measurements and 21,908 SK‐MEL‐2 measurements.

Differences in the geometric and positional characteristics of the melanocytes and SK‐MEL‐2 cells are represented by the violin plots in Figure 5. The figures reveal that SK‐MEL‐2 cells may overwhelmingly be characterized by greater variation per parameter measured. For the boxed center position X and Y, SK‐MEL‐2 cells are characterized by greater median values compared to the melanocytes, suggesting that the geometric center of SK‐MEL‐2 cells lies at greater values (Figure 5A,B). There is more variation in the position of the geometric center for SK‐MEL‐2 cells than melanocytes, given their more polarized distribution. The peak position X and Y displayed similar patterns and overlapping values as the boxed center position X and Y for both cell lines, with SK‐MEL‐2 cells bearing greater distributions and median values in contrast to the melanocytes (Figure 5E,F). This suggests that SK‐MEL‐2 cells have a more diverse range of coordinates with which the thickest location was measured. The centroid position X and Y revealed similar patterns, in which SK‐MEL‐2 cells continued to show greater variation in their distribution (Figure 5C,D). The melanocytes tended to be more concentrated around their median values, suggesting greater cohesiveness in the location of the center of mass for melanocytes than SK‐MEL‐2 cells. Statistical significance was exhibited across all of the geometric and positional parameters. The mean values listed in Table 1 were consistent with the median values displayed in the violin plots, indicating that outliers did not interfere with the overall trends for the positional and geometric characteristics. In addition, the mean values show great similarity in the X and Y coordinates for the boxed center and peak positions, suggesting that the geometric center and the thickest part of the cells tend to coincide with one another for both cell lines.

### 3.6 SK‐MEL‐2 Cells Are Characterized by Greater Phaseshift Values, Fewer Variations in Height

**Figure 6:**
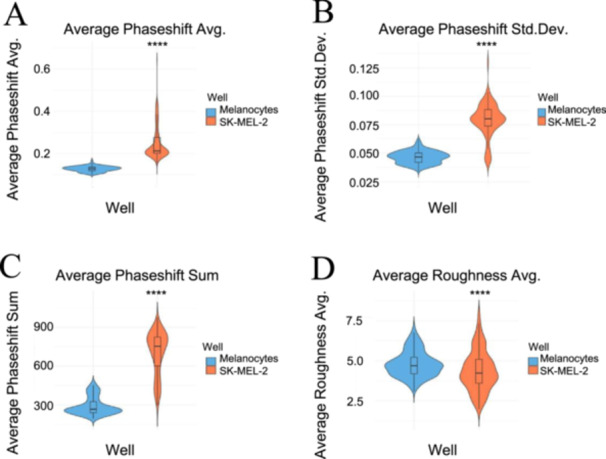
Violin plots of phaseshift and roughness parameters for melanocytes versus SK‐MEL‐2 cells. The SK‐MEL‐2 cells exhibited greater, more distributed values for (A) phaseshift avg, (B) phaseshift std. dev, and (C) phaseshift sum, indicating greater phase shifts. The melanocytes exhibited greater (D) roughness avg, suggesting rougher surfaces compared to the SK‐MEL‐2 cells. Melanocytes are represented in the color blue, and SK‐MEL‐2 cells are visualized in the color orange. The plots were generated in R (version 4.4.0), and the statistical significance was indicated as **** for *p* < 0.0001 comparing the two cell lines. Analysis included 21,908 melanocyte measurements and 21,908 SK‐MEL‐2 measurements.

Melanocytes and SK‐MEL‐2 cells revealed distinct patterns with respect to their phaseshift and roughness parameters, suggesting differences in internal composition between the two cell lines. To begin, the two cell lines differed greatly in their phaseshift parameters, including phaseshift average, standard deviation, and sum, as revealed in Figure 6A–C. Specifically, SK‐MEL‐2 cells had greater distributions and higher median values in contrast to the melanocytes, which were characterized by lower values more concentrated around their medians. This reveals that the SK‐MEL‐2 cells had greater phase shifts measured within the cell areas, suggesting that there are differences in internal composition compared to the melanocytes.

Roughness average, on the other hand, revealed opposite patterns (Figure 6D), with melanocytes being characterized by greater median values. However, similar to the phaseshift parameters, the melanocyte roughness values were more concentrated toward their median as opposed to the SK‐MEL‐2 cells, which revealed greater variation in their distribution. These findings suggest that melanocytes are more uniform in their surface roughness values, having rougher surfaces on average.

These findings align with the mean values listed in Table 1, in which the mean values of SK‐MEL‐2 cells for the phaseshift parameters are higher than those of the melanocytes. Likewise, the mean values of the roughness average displayed a similar trend to that shown in the violin plot, where the melanocytes had higher values than the SK‐MEL‐2 cells. This consistency between the median and mean patterns signifies that the presence of any outliers did not greatly influence the patterns shown in the violin plots. Statistical significance was observed for all of the parameters, indicating potential as a biomarker for cell differentiation.

### 3.7 Texture Parameters Highlight Great Differences and Variability for Both Melanocytes and SK‐MEL‐2 Cells

**Figure 7:**
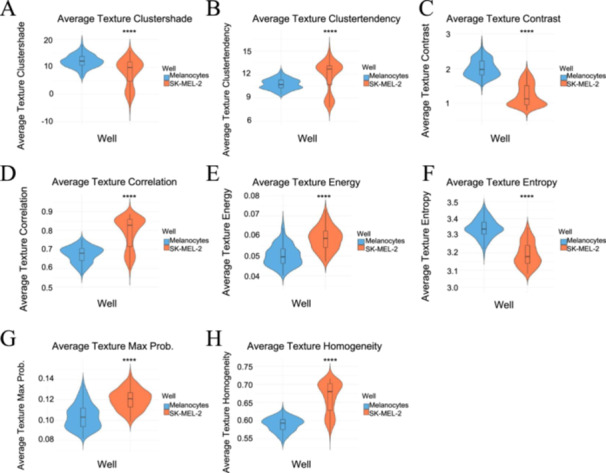
Violin plots of texture parameters for melanocytes versus SK‐MEL‐2 cells. The melanocytes exhibited greater, more concentrated values in (A) texture clustershade, but lower values in (B) texture clustertendency. The SK‐MEL‐2 cells exhibited lower (C) texture contrast but greater (D) texture correlation and (E) texture energy, suggesting greater uniformity in image texture. Additionally, the melanocytes exhibited greater (F) texture entropy but smaller (G) texture max. prob. and (H) texture homogeneity, suggesting more melanocyte nonuniformity and complexity. The plots were visualized in R (version 4.4.0), with melanocytes in blue and SK‐MEL‐2 cells in orange. Statistical significance was indicated as **** for *p* < 0.0001 comparing the two cell lines. Analysis included 21,908 melanocyte measurements and 21,908 SK‐MEL‐2 measurements.

Texture parameters are highlighted in the violin plots in Figure 7, revealing distinct patterns between the melanocytes and SK‐MEL‐2 cells.

The two cell lines varied greatly in the first set of texture parameters, including texture clustershade, clustertendency, contrast, and correlation, as illustrated in Figure 7A–D. With respect to texture clustershade and texture contrast, melanocytes exhibited greater medians in comparison to the SK‐MEL‐2 cells; these patterns suggest that the melanocytes were less symmetrical than the SK‐MEL‐2 cells with a greater degree of depth variation. On the other hand, SK‐MEL‐2 cells had greater values for texture clustertendency and texture correlation, indicating a greater consistency and uniformity in image texture. Largely among the parameters in this first set, SK‐MEL‐2 cells consistently had greater distributions than the melanocytes, revealing that they tended to have more variety in their texture.

There is similar variability in the second set of texture parameters, which includes texture energy, entropy, maximum probability (max. prob.), and homogeneity (Figure 7E–H). The SK‐MEL‐2 cells revealed greater median values for texture energy, max. prob., and homogeneity, revealing greater texture nonrandomness in comparison to the melanocytes. Melanocytes, on the other hand, had a higher texture entropy, revealing a greater nonuniformity and complexity in comparison to the SK‐MEL‐2 cells. The mean values of the texture properties for both melanocytes and SK‐MEL‐2 cells listed in Table 1 were consistent with the trends observed in the violin plots. This suggests that potential outliers did not skew the trends displayed. Statistical significance was observed in regard to all of the parameters, suggesting potential in differentiating and classifying the two cell lines.

### 3.8 Ranked Feature Importance Reveals Optical Parameters as Most Significant in Differentiating Melanocytes and SK‐MEL‐2 Cells

**Figure 8:**
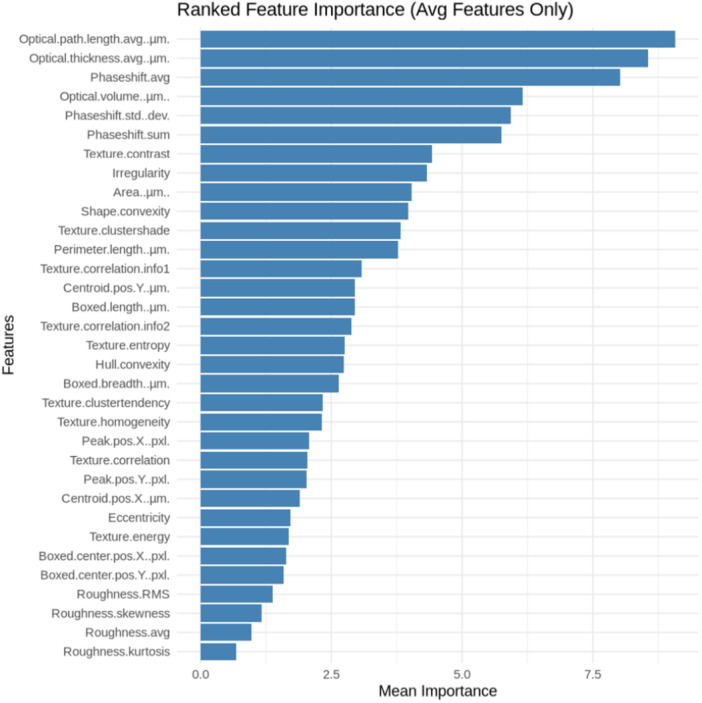
Ranked Feature Importance Diagram in differentiating melanocytes from SK‐MEL‐2 cells based on the Random Forest model. The Ranked Feature Importance Diagram reveals the parameters most significant in differentiating between the melanocytes and SK‐MEL‐2 cells. The optical parameters were demonstrated as the most significant, while the roughness parameters were the least significant. The diagram was generated using R (version 4.0.5). Analysis included 21,908 melanocyte measurements and 21,908 SK‐MEL‐2 measurements.

The Ranked Feature Importance Plot, shown in Figure 8, was utilized to elucidate which of the cellular properties analyzed are most significant in differentiating between the melanocytes and SK‐MEL‐2 cells. Specifically, the greater the feature importance, the better the Random Forest model was able to distinguish between the cell types. The plot illustrates the optical parameters as being of most significance in this differentiation, including optical path length, optical thickness avg., optical volume, and phaseshift avg. On the contrary, the Roughness parameters were labeled as least significant. These findings suggest the potential for the optical differences between melanocytes and SK‐MEL‐2 cells to be used as biomarkers to characterize cancer development.

### 3.9 SK‐MEL‐2 Cells Exhibit Increases in Mean Cell Area, Volume, and Diameter Over 48 h, While Melanocytes Remain Relatively Consistent

**Figure 9:**
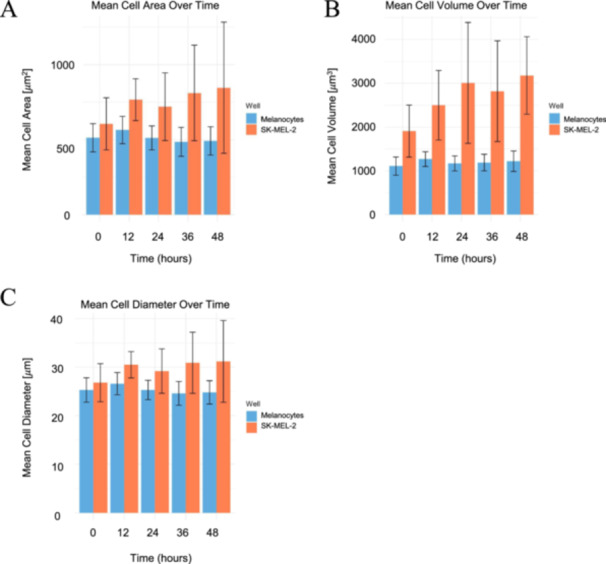
Mean area, volume, and diameter of melanocytes versus SK‐MEL‐2 cells over 48 h. Bar graphs highlighted the general increase in SK‐MEL‐2 (A) mean cell area, (B) mean cell volume, and (C) mean cell diameter at 0, 12, 24, 36, and 48 h, while the melanocytes remained relatively consistent. Melanocytes are represented in the color blue, and SK‐MEL‐2 cells are visualized in the color orange. The plots were generated in R (version 4.4.0). Error bars represent the standard deviation of the measurements, exhibiting variability and possible significance across the parameters measured. Analysis included 21,908 melanocyte measurements and 21,908 SK‐MEL‐2 measurements.

The melanocytes and SK‐MEL‐2 cells exhibited variations in their mean cell area, volume, and diameter over the course of 48 h, with data measurements obtained at 12‐h intervals (Figure 9). With regard to mean cell area (Figure 9A), at Hour 0, melanocytes started at 513 µm^2^, slightly decreasing to 492 µm^2^ at Hour 48. SK‐MEL‐2 cells, on the other hand, exhibited an increase in mean cell area, beginning at 606 µm^2^ and ending with a mean cell area of 846 µm^2^ at Hour 48. Standard deviation error bars are displayed in the graph, indicating variations in data values. When comparing the two cell lines, SK‐MEL‐2 consistently displayed greater mean cell area values over time.

Similarly, the melanocytes and SK‐MEL‐2 cells revealed distinct trends in mean cell volumes over time (Figure 9B). The melanocytes began with a mean cell volume of 1110 µm^3^ at Hour 0, increasing slightly to 1220 µm^3^ at Hour 48. In contrast, SK‐MEL‐2 cells began with a mean cell volume of 1910 µm^3^ at Hour 0 and increased to 3180 µm^3^ at Hour 48. Similar to the melanocytes, SK‐MEL‐2 cells exhibited an increase in mean cell volume; however, SK‐MEL‐2 cells displayed a more prominent increase over time, while the increase was more modest for melanocytes. The error bars exhibit a significant difference between melanocyte and SK‐MEL‐2 mean cell volume at Hours 12 through 48.

Furthermore, the melanocyte and SK‐MEL‐2 mean cell diameter showed distinct trends (Figure 9C). The melanocytes started with a mean cell diameter of 25.3 µm at Hour 0, decreasing to 24.8 µm at Hour 48. SK‐MEL‐2 cells, on the contrary, began with a mean cell diameter of 26.8 µm, increasing to 31.2 µm at Hour 48. The error bars indicate the standard deviation of the mean cell diameter measurements. Overall, the melanocytes presented a relatively consistent mean cell volume, while SK‐MEL‐2 cells had a slight increasing trend.

## 4 Discussion

The melanocyte and SK‐MEL‐2 cellular data extracted from holographic microscopy denote the differences between the two cell lines across various parameters, including morphology, geometry, and optical properties.

To begin, the 2D and pseudo‐3D holographic microscopy of the melanocytes and SK‐MEL‐2 cells provided insights into their overt morphologies. As demonstrated in Figure 1A, the melanocytes were characterized by long branches that extended from the cell body, with irregular boundaries. Previous studies have found that melanocytes extend their branches to interact with other cells, including the Langerhans cells of the immune system, and to transport melanosomes to keratinocytes [8, 33, 34]. The SK‐MEL‐2 cells, however, were more polygonal in shape, with shorter arm extensions. Short dendrites have been found in some instances of nodular melanoma, which is the most aggressive form of melanoma [7]. Further studies may be conducted to determine the role of the dendrites in *NRAS*‐mutant SK‐MEL‐2 cells compared to healthy melanocytes and its possible implication in cancerous states. In addition, the 2D microscopy revealed that both melanocytes and SK‐MEL‐2 cells are elongated. The pseudo‐3D holographic microscopy corroborated the findings from the 2D microscopy via single‐cell analysis and comparison (Figure 1B). The SK‐MEL‐2 cells were notably thicker, with more polygonal bodies than the melanocytes. These findings are aligned with the violin plots measuring morphology, as demonstrated across Figures 4, 5, 6, 7.

Furthermore, the validity of the characteristics used to compare the two cell lines was examined in Figure 2 via a K‐Means Elbow Clustering Plot as well as a Hierarchical Clustering Plot. Despite using different methods, both plots characterize data sets into distinct clusters based on similarities between the data points. Both demonstrate a clear separation between the melanocytes and SK‐MEL‐2 cells based on the morphological data sets, suggesting that these parameters capture significant biological differences between the two cell lines.

Additionally, the results illustrated in the PCA and t‐SNE Plot demonstrate a clear separation between the melanocytes and SK‐MEL‐2 cells (Figure 3). The PCA Plot illustrates a compact cluster of melanocyte data points and a broader range of SK‐MEL‐2 data points. These differences in shape highlight the greater homogeneity in melanocyte characteristics, in contrast to the more heterogeneous SK‐MEL‐2 pool of characteristics. In addition, the distinct boundary between the melanocytes and SK‐MEL‐2 cells illustrates that between the melanocytes and SK‐MEL‐2 cells, there exists clear differences between them. The t‐SNE Plot expands on these findings from the PCA Plot; it showcases that there is still dissimilarity among the melanocytes themselves. It also corroborates the findings from the PCA Plot that the SK‐MEL‐2 cell characteristics exhibit distinct patterns from melanocytes. The PCA and t‐SNE plots, overall, provided an overarching view of the distinctions between melanocytes and SK‐MEL‐2 cells.

A much deeper analysis of the specific differences between the melanocytes and SK‐MEL‐2 cells is illustrated by violin plots (Figures 4, 5, 6, 7). To begin, the violin plots in Figure 4 compare the morphological and optical features of melanocytes and SK‐MEL‐2 cells. This analysis demonstrates the overwhelming homogeneity in melanocyte morphology compared to SK‐MEL‐2 morphology: SK‐MEL‐2 cells had wider distributions in their characteristics in contrast to the melanocytes. The melanocytes exhibited smaller areas, dimensions, optical volumes, and perimeter lengths in comparison to the SK‐MEL‐2 cells. Despite the variations in these parameters measured over 48 h (Figure 9), the melanocytes consistently exhibited smaller values than the SK‐MEL‐2 cells. Over time, the mean cell area and mean cell diameter of the melanocytes remained relatively consistent, though slightly decreased, whereas the SK‐MEL‐2 cells exhibited an increase in mean cell area over time. In contrast, the mean cell volume increased over time for both melanocytes and SK‐MEL‐2 cells, though to different degrees. Other morphological patterns revealed that melanocytes and SK‐MEL‐2 cells are similarly elongated, though the melanocytes displayed a more irregular circumference with fewer changes in optical thickness (Figure 4). These findings are corroborated by the mean values illustrated in Table 1.

To our knowledge, other studies have not directly compared the morphologies of melanocytes and SK‐MEL‐2 cells; nonetheless, prior research has characterized SK‐MEL‐2 cells with an elongated, polygonal morphology [26], and melanocytes have been characterized with oval, fusiform, or dendritic shapes [27]. Our findings align with these conclusions, as in our study, SK‐MEL‐2 cells exhibited polygonal morphologies, while the melanocytes had irregular, dendritic shapes.

A further analysis comparing the two cell lines is denoted by the violin plots measuring positional and geometric parameters (Figure 5). Even though the melanocytes and SK‐MEL‐2 cells had different geometric coordinates in every aspect, their geometric centers overlapped with their thickest measured location; thus, although their geometric coordinates differed, they still revealed similar trends. They also had different centers of mass, suggesting differences in the internal structure of melanocytes and SK‐MEL‐2 cells. In addition, the differences in phaseshift and roughness values further suggest variations in the internal composition between the two cell lines given SK‐MEL‐2 cells were characterized by greater phaseshift values as well as fewer variations in height (Figure 6). Such findings emphasize the unique features of each cell line.

Variations also existed between the melanocytes and SK‐MEL‐2 cells in terms of their texture parameters, as demonstrated by Figure 7. Overall, the trends revealed that the melanocytes had a less symmetrical image and a greater degree of depth variation; however, SK‐MEL‐2 cells had greater consistency, nonrandomness, and uniformity in image texture. Lastly, in Figure 8, the Ranked Feature Importance Plot reveals optical parameters as most significant in differentiating between the two cell lines, whereas the roughness measurements were the least significant.

Since we did not measure directly the refractive index of the cells, all thickness‐ and volume‐related parameters reported here represent relative optical quantities derived from quantitative phase reconstructions [59, 60]. Previous optical studies have shown that refractive indices vary among different cell types [61, 62], which could influence apparent optical thickness and volume [60, 63]. Future work combining DHM with refractive‐index tomography could further refine these measurements and provide more precise physical estimates.

In conclusion, this exploration into the differences between melanocytes and SK‐MEL‐2 cells aids in further defining the two cell lines in terms of morphological, optical, geometric, phaseshift, and texture parameters. Further research could be conducted to understand the differences in cell motility and migration between melanocytes and SK‐MEL‐2 cells. In addition, *NRAS*‐mutant melanoma could be compared with other mutated melanomas to better understand the nature of this cancer and the characteristics conferred by different mutations. Our findings, along with any further research into the matter, would aid in further characterizing melanoma, and they could potentially introduce new biomarkers to differentiate the two cell lines via holographic microscopy.

## Conflicts of Interest

The authors declare no conflicts of interest.

## Data Availability

The quantitative phase imaging data sets and derived morphological feature tables generated for this study are available from the corresponding author on reasonable request.
